# Acquired bedaquiline resistance during the treatment of drug-resistant tuberculosis: a systematic review

**DOI:** 10.1093/jacamr/dlac029

**Published:** 2022-03-29

**Authors:** Jahan Saeed Mallick, Parvati Nair, Elizabeth Tabitha Abbew, Armand Van Deun, Tom Decroo

**Affiliations:** 1 Institute of Tropical Medicine Antwerp, Department of Clinical Sciences, Kronenburgstraat 43, 2000 Antwerpen, Belgium; 2 Cape Coast Teaching Hospital, Interberton Road, Cape Coast, Ghana; 3 Independent consultant, Leuven, Belgium

## Abstract

**Background:**

Drug-resistant tuberculosis (DR-TB) is considered to be a public health threat and is difficult to cure, requiring a lengthy treatment with potent, potentially toxic drugs. The novel antimicrobial agent bedaquiline has shown promising results for patients with DR-TB, improving the rate of culture conversion and reducing TB-related mortality. However, increasing numbers of cases with acquired bedaquiline resistance (ABR) have been reported in recent years.

**Methods:**

This systematic review aimed to assess the frequency of ABR and characteristics of patients acquiring it. Studies showing data on sequential bedaquiline drug-susceptibility testing in patients treated with a bedaquiline-containing regimen were included. The databases CENTRAL, PubMed and Embase were manually searched, and 866 unique records identified, eventually leading to the inclusion of 13 studies. Phenotypic ABR was assessed based on predefined MIC thresholds and genotypic ABR based on the emergence of resistance-associated variants.

**Results:**

The median (IQR) frequency of phenotypic ABR was 2.2% (1.1%–4.6%) and 4.4% (1.8%–5.8%) for genotypic ABR. Among the studies reporting individual data of patients with ABR, the median number of likely effective drugs in a treatment regimen was five, in accordance with WHO recommendations. In regard to the utilization of important companion drugs with high and early bactericidal activity, linezolid was included in the regimen of most ABR patients, whereas the usage of other group A (fluoroquinolones) and former group B drugs (second-line injectable drugs) was rare.

**Conclusions:**

Our findings suggest a relevant frequency of ABR, urging for a better protection against it. Therefore, treatment regimens should include drugs with high resistance-preventing capacity through high and early bactericidal activity.

## Introduction

Tuberculosis (TB) is an infectious disease caused by the bacterium *Mycobacterium tuberculosis*. It is the second leading cause of death from a single infectious agent worldwide after COVID-19.^1^ Particularly, rifampicin-resistant TB (RR-TB) is considered to be a public health threat and is difficult to cure, requiring a lengthy treatment with several potent, potentially toxic drugs. According to WHO, an estimated global total of 465* *000 people fell ill with RR-TB or MDR-TB in 2019.^2^

In 2012 the US FDA granted accelerated approval to the antitubercular agent bedaquiline, the first new anti-TB drug after rifampicin, which was released almost 40 years earlier. Its mechanism of action is the targeting and inhibition of the mycobacterial enzyme ATP synthase.^3^ When testing its efficacy in clinical trials, it was shown to increase the rate of culture conversion and cure compared with a background regimen with placebo.^4^ It could be demonstrated that its use improves treatment outcomes significantly.^5^ Additionally, its inclusion in a treatment regimen was associated with a 3-fold reduction in mortality of patients with MDR/RR-TB and an even larger one for patients with additional resistance to fluoroquinolones (FQs) and at least one of the second-line injectable drugs (SLIDs).^6,7^ Available clinical data from all over the world support a good safety and tolerability profile of bedaquiline.^8^

Since 2018 WHO has recommended using bedaquiline as a core drug in patients with MDR/RR-TB.^7^ One year later WHO advised discontinuing the implementation of injectable-containing regimens for drug-resistant TB (DR-TB) and making the short all-oral bedaquiline-containing regimens the preferred treatment choice.^9^ By the end of 2020, 109 countries worldwide were using bedaquiline for their citizens infected with TB.^1,2^ Besides standardized short all-oral regimens, it can also be administered as part of individualized longer regimens composed based on patient characteristics following the WHO grouping (A, B, C) of anti-TB drugs or under operational research conditions.^7^

Naturally, the widespread use of a new antibacterial drug comes with the risk of emerging resistance. As of today, several genetic mutations or resistance-associated variants (RAVs) have been linked with bedaquiline resistance, with *Rv0678* and *atpE* being the most important ones.^10^*Rv0678* codes for the MmpR transcriptional repressor of the MmpS5-MmpL5 efflux system and its mutations are usually associated with low-level bedaquiline resistance, also conferring cross-resistance to clofazimine and azoles.^11,12^ The gene *atpE* encodes the ATP synthase subunit C. Mutations confer high-level bedaquiline resistance, but their frequency is relatively low among patients with TB.^11^ Additionally, it has been demonstrated that mutations in the gene *pepQ*, encoding an aminopeptidase, can cause low-level resistance to bedaquiline and clofazimine.^13^ Furthermore, genetic alterations in *Rv1979c*, encoding an uncharacterized transporter, have been described, but do not lead to relevant increases of bedaquiline MICs in the vast majority of cases.^10^ Nevertheless, there is no comprehensive register of RAV subtypes available, as there is only limited knowledge about the level of phenotypic resistance each mutation confers.

Even though once established antibiotic resistance is usually transmitted, acquired antimicrobial resistance also contributes to the spread of DR-TB.^14^ In the case of acquired bedaquiline resistance (ABR), mutants do not seem to suffer any fitness costs in comparison to their isogenic wild types.^15^ Therefore, it is not surprising that ABR is correlated with adverse treatment outcomes.^16–19^ Several factors can enhance the development of ABR. One main aspect is resistance to other drugs of the regimen, particularly FQs. The bactericidal activity of bedaquiline is relatively weak in the first days and takes about 1 week to develop.^20–22^ To prevent the selection of core drug-resistant mutants during the first treatment days, high early bactericidal activity of companion drugs is needed to reduce the bacillary load.^23^ It is expected that this role would be fulfilled by the FQs in the majority of cases. However, the global prevalence of FQ-resistant MDR/RR-TB cases in the last 15 years was 20.1% and only just over 50% of MDR/RR-TB cases were tested for FQ resistance.^1,2^ In case of undetected or low-level FQ-resistant strains, it can lead to the selection of those resistant mycobacteria, even if only present as a subgroup [coexisting susceptible and resistant microorganisms of one (heteroresistance) or more than one strain (mixed infection)]. By the time bedaquiline reaches its full bactericidal capacity, it is no longer protected by the FQ, consequently resulting in a considerable risk of ABR, particularly when the regimen does not include any other drug with a high early bactericidal effect.^21^ Even though WHO recommends drug-susceptibility testing (DST) at least for FQs before bedaquiline initiation, they also recognize the limited feasibility in many settings, especially where resources are scarce.^7^

There are other factors to be considered when investigating potential causes of ABR. Bedaquiline has a long terminal elimination half-life of 5.5 months, most likely due to the slow release of the drug and its metabolites from peripheral tissues.^24^ Hence, early treatment discontinuation or prolonged exposure can lead to persistent low plasma levels, while other drugs with shorter half-lives would be cleared and therefore cannot protect against ABR.^24,25^ Also, its hepatic metabolism may cause drug–drug interactions, especially with antiretrovirals. It could be demonstrated that efavirenz reduces steady-state concentrations of bedaquiline and its metabolites through induction of CYP3A4. As this leads to subtherapeutic levels of bedaquiline, it could play a role in the development of ABR.^26^ Conversely, other anti-HIV drugs such as ritonavir (e.g. in lopinavir/ritonavir, darunavir/ritonavir) act as CYP3A4 inhibitors, resulting in increased bedaquiline levels and therefore could potentially increase its bactericidal effect.^27,28^ No interactions with bedaquiline are anticipated with the recently introduced antiretroviral core drug dolutegravir.^29^ Additionally, there seem to be pharmacogenetic elements involved in bedaquiline metabolism, as its clearance is around 52% higher in black patients.^30^ Another challenge is cross-resistance. For instance, *Rv0678* mutations can confer cross-resistance to clofazimine and azoles, as mentioned earlier.^31^

Yet, the frequency of ABR remains unclear. For further investigation, Tahseen and colleagues^21^ analysed three recent cohort studies of DR-TB patients treated with bedaquiline-containing regimens and found an ABR frequency that ranged between 2.5% and 30.8%. However, to our knowledge there is no review of the available literature that assesses the extent of ABR, which is the aim of this systematic review.

## Materials and methods

### Preparation

A classic research protocol was not developed. However, a concept based on the PRISMA guidelines and the Cochrane Handbook for Systematic Reviews of Interventions was established before starting data retrieval and analysis.^32,33^ The goal to register this study on PROSPERO could not be met, as only COVID-19-related systematic reviews were accepted when this review started.^34^

### Search strategy

This systematic review aimed to estimate the frequency of ABR during the treatment with bedaquiline-containing regimens among patients with DR-TB. Apart from the frequency of ABR, trial characteristics, treatment regimens and outcomes as well as certain features of patients acquiring bedaquiline resistance were assessed and analysed.

The three databases CENTRAL, PubMed and Embase were searched using specific search terms on 7 February 2021.^33^ Additionally, the references of included studies were checked for eligibility. Study data were extracted to an Excel worksheet (Microsoft Office Standard 2019) and duplicates were removed manually. The software Citavi (Version 6.7) was used for managing references. The full search strategy is illustrated in Table S1 (available as Supplementary data at *JAC-AMR* Online).

### Eligibility criteria and study selection

After duplicate removal, title and abstracts of the individual records were screened and studies excluded if they did not meet the eligibility criteria. As a second step, the full text of remaining articles was assessed based on the inclusion and exclusion criteria (Table S1). The study selection was executed independently by two reviewers (J.S.M. and P.N.) and disagreements were solved through discussion or by involvement of a third reviewer (E.T.A.). Original studies of DR-TB patients infected with *M. tuberculosis* treated with bedaquiline-containing regimens and sequential DST for bedaquiline were included in this review. Those not clearly indicating which participants were treated with bedaquiline, solely reporting on patients with bedaquiline resistance, with an average bedaquiline exposure for less than 6 months or that were not available in the English language were excluded from this analysis. The PRISMA flow chart was illustrated with the freeware draw.io (https://app.diagrams.net).^32^

### Data extraction

Data were extracted to Excel worksheets. The variables of interest were study location, type, duration, DR-TB cohort, frequent comorbidities (HIV, hepatitis C) and TB treatment history, treatment regimen, bedaquiline exposure, outcome, number of patients treated with bedaquiline, number of patients with baseline and sequential bedaquiline DST and number of patients with baseline and acquired bedaquiline resistance. Furthermore, information on individual data of patients with ABR (e.g. treatment regimens, resistance patterns, type and time of appearance of ABR, MIC changes) was obtained and summarized in a table inspired by the reporting of Tahseen and colleagues.^21^ The WHO 2020 classification of resistance patterns was applied and ‘pre-XDR’ was defined as MDR-TB with additional drug resistance to any FQ or SLID.^7^ If needed, study authors were contacted for clarification.

### Data synthesis

Descriptive statistical measures such as proportions and percentages as well as medians, ranges and IQRs were used to summarize the extracted data for patients treated with bedaquiline-containing regimens. No meta-analysis was performed because of the heterogeneity of included studies. Individual patient data was collected from a subgroup of patients with ABR for further analysis. Variables of interest were resistance pattern, average number of likely effective drugs and use of current group A (FQs, linezolid) and former group B drugs (SLIDs) to describe the proportion with either a favourable or unfavourable outcome stratified by these factors among patients who acquired bedaquiline resistance. WHO defines the likelihood of effectiveness of a drug based on proven susceptibility, no resistance to another drug with cross-resistance, rare use or a low level of drug resistance in the setting and no previous use in a failing regimen.^35^ As the individual patient data provided by study authors were limited, the main criteria for the consideration as likely effective were proven susceptibility, no evidence of (cross-)resistance or no previous use. Data extraction and synthesis were performed individually by two reviewers (J.S.M. and E.T.A.) and any disagreements were settled by discussion or inclusion of a third reviewer (P.N.).

### ABR

ABR describes phenotypic or genotypic resistance to bedaquiline that emerged during treatment of patients with documented susceptibility at baseline.^10^ Not all studies provided data on bedaquiline MICs. Therefore, ABR was assessed separately based on MIC thresholds and evolution and appearance of bedaquiline RAVs.

Coherent with the results of the multi-country, multi-laboratory bedaquiline MIC validation study by Kaniga *et al*.,^36^ an MIC susceptibility breakpoint of 0.12 mg/L for the Middlebrook 7H9 broth microdilution method (7H9) and critical concentrations of 0.25 mg/L for the Middlebrook 7H11 agar proportion (7H11) and 1 mg/L for the mycobacteria growth indicator tube (MGIT) methods were applied. This resulted in susceptibility thresholds of <0.25 mg/L (7H9/7H11) and <1 mg/L (MGIT) and as a consequence assumed resistance above these levels. Patients with MIC levels above these thresholds before treatment start were considered having phenotypic baseline bedaquiline resistance. In patients with DST showing bedaquiline susceptibility at baseline, phenotypic ABR was assessed using the same thresholds. Moreover, following the approach of Tahseen *et al*.,^21^ MIC increases that were at least 4-fold but not lower than 0.12 mg/L (7H9/7H11) or 0.5 mg/L (MGIT) were also presumed as phenotypic ABR.

Due to the limited knowledge about bedaquiline RAVs and their non-standardized reporting, the appearance of any new RAV mutations in *Rv0678*, *atpE* or *pepQ* in sequential isolates were considered as an indicator of genotypic ABR.^37^ Patients expressing these RAVs before treatment initiation were regarded as having genotypic baseline bedaquiline resistance. As *Rv1979c* mutations do normally not lead to relevant MIC increases, they were not assumed to be suggestive of genotypic ABR in this analysis.^10^

Four measures of ABR were calculated (Table S2). For the first two measures, the calculation of the frequency of ABR, the number of patients with ABR was used as the numerator based on MIC increase (phenotypic ABR) and appearance of bedaquiline RAVs (genotypic ABR), respectively. For two additional measures the numerator only included those with both ABR and a clinically adverse outcome (treatment failure, death, loss to follow-up).

### Qualitative assessment

The Newcastle-Ottawa Scale was applied to assess the quality of the included cohort studies.^38^ For the criterion ‘demonstration that the outcome of interest was not present at start of the study’ bedaquiline DST needed to be performed for at least 80% of the participants treated with bedaquiline. An adequate follow-up time was defined as a period of 6 months after the end of bedaquiline treatment. This was based on the long terminal elimination half-life of bedaquiline (5.5 months) and the potential late emergence of resistance after exposure to bedaquiline.^24^ Cohort studies were rated as having low, medium or high risk of bias if they were given ≥7, 4–6 or ≤3 stars, respectively. For the remaining two studies the revised Cochrane risk-of-bias tool for randomized trials (RoB 2) was used.^39^ Assessment was performed independently by two reviewers (J.S.M. and E.T.A.) and disagreements were solved by involvement of a third reviewer (P.N.). Tables for illustration were constructed with the software PowerPoint (Microsoft Office Standard 2019).

## Results

### Study selection

**Figure 1.**
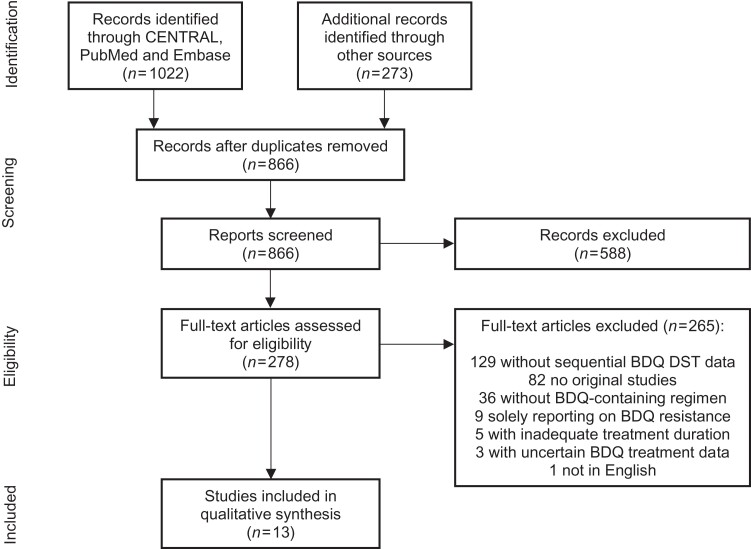
PRISMA flow chart. Chart showing the number of studies included for assessing the frequency of ABR. BDQ, bedaquiline.

The search identified 866 unique papers of which 588 records were excluded by title and abstract screening. The full text of 278 articles was assessed for eligibility, leading to the inclusion of 13 studies (Figure 1).

### Study characteristics

Of all 13 studies included in this review, 11 were cohort studies (5 prospective, 6 retrospective) while 2 were randomized controlled trials. Ten were based at a single centre and three were performed at multiple sites. Eight studies reported initial resistance patterns of their participants treated with bedaquiline. Five of those mainly included participants with advanced drug-resistance (pre-XDR- and XDR-TB), whereas patients with MDR-TB formed the biggest cohort in three studies. Comorbidities were only infrequently reported. The prevalence of HIV ranged from 3.1%–100.0%, that of hepatitis C ranged from 20.3%–46.7% (based on serology) and that of having a history of TB treatment ranged from 0.0%–90.9% (Table 1).

**Table 1.** Characteristics of included studies

Study	Location	Type	DR-TB cohort^a^, % (*n*/*N*)	Comorbidities^b^, % (*n*/*N*)	Duration
Conradie *et al*. 2020^57^	South Africa	Prospective cohort study	MDR: 34.9 (38/109)	HIV: 51.4 (56/109)	Apr 2015–Nov 2017
			pre-XDR: 0.0 (0/109)	HCV: N/A	
			XDR: 65.1 (71/109)	TBH: complicated MDR-TB^c^	
Ghodousi *et al*. 2019^16^	Pakistan	Retrospective cohort study	MDR: 26.7 (8/30)	HIV: N/A	Nov 2017–May 2018
			pre-XDR: 50.0 (15/30)	HCV: N/A	
			XDR: 23.3 (7/30)	TBH: N/A	
Liu *et al*. 2021^58^	China	Retrospective cohort study	MDR: N/A	HIV: N/A	N/A
			pre-XDR: N/A	HCV: N/A	
			XDR: N/A	TBH: N/A	
Nimmo *et al*. 2020^18^	South Africa	Prospective cohort study	MDR: N/A	HIV: 100.0 (92/92)	Nov 2016–Jan 2019
			pre-XDR: N/A	HCV: N/A	
			XDR: N/A	TBH: 66.0 (61/92)	
Nimmo *et al*. 2020^47^	South Africa	Retrospective cohort study	MDR: N/A	HIV: N/A	N/A
			pre-XDR: N/A	HCV: N/A	
			XDR: N/A	TBH: N/A	
Diacon *et al*. 2014^59^	Brazil, India, Latvia, Peru, the Philippines, Russia, South Africa, Thailand	Randomized placebo-controlled trial, double-blind	MDR: N/A	HIV: N/A	N/A
			pre-XDR: N/A	HCV: N/A	
			XDR: N/A	TBH: 0.0 (0/79) with DR-TB treatment history	
Pym *et al*. 2016^19^	China, South Korea, the Philippines, Thailand, Estonia, Latvia, Russia, Turkey, Ukraine, Peru, South Africa	Prospective cohort study	MDR: 60.5 (124/205)	HIV: N/A	Aug 2009–Sep 2010
			pre-XDR: 21.5 (44/205)	HCV: N/A	
			XDR: 18.0 (37/205)^d^	TBH: 94.1 (193/205)^d^	
Guglielmetti *et al*. 2017^60^	France	Retrospective cohort study	MDR: 22.2 (10/45)	HIV: 4.4 (2/45)	Jan 2011–Dec 2013
			pre-XDR: 24.5 (11/45)	HCV: 46.7 (21/45)	
			XDR: 53.3 (24/45)	TBH: 75.6 (34/45)	
Guglielmetti *et al*. 2018^61^	France, Latvia	Retrospective cohort study	MDR: 0.0 (0/10)	HIV: N/A	Jan 2013–Aug 2015
			pre-XDR: 40.0 (4/10)	HCV: N/A	
			XDR: 60.0 (6/10)	TBH: N/A	
Diacon *et al*. 2012^24^	South Africa	Randomized placebo-controlled trial, double-blind	MDR: 91.4 (21/23)	HIV: 13.0 (3/21)^e^	N/A
			pre-XDR: 4.3 (1/23)	HCV: N/A	
			XDR: 4.3 (1/23)	TBH: N/A	
Kempker *et al*. 2020^62^	Georgia	Prospective cohort study	MDR: 81.2 (52/64)	HIV: 3.1 (2/64)	Nov 2016–Jan 2019
			pre-XDR: 0.0 (0/64)	HCV: 20.3 (13/64)	
			XDR: 18.8 (12/64)	TBH: 50.0 (32/64)	
Mokrousov *et al*. 2021^42^	Russia	Retrospective cohort study	MDR: 9.1 (1/11)	HIV: 45.5 (5/11)	2018–19
			pre-XDR: 9.1 (1/11)	HCV: N/A	
			XDR: 81.8 (9/11)	TBH: 90.9 (10/11)	
Nimmo *et al*. 2020^63^	South Africa	Prospective cohort study	MDR: N/A	HIV: N/A	2016–19
			pre-XDR: N/A	HCV: N/A	
			XDR: N/A	TBH: N/A	

HCV, HCV positive; N/A, not available; TBH, TB history.

a Following the WHO 2020 definitions of resistance patterns. Pre-XDR was defined as MDR-TB with additional drug resistance to any FQ or SLID.^7^

b HIV, hepatitis C, TB treatment history.

c Complicated MDR-TB: only MDR-TB patients who were non-responsive to first-line treatment or for whom it was stopped because of side effects were included.

d Data for the modified intention-to-treat population.

e Only available for the 21 patients included in the efficacy analysis.

### Treatment regimens and outcomes

In most studies bedaquiline-containing regimens were individualized; only one study used a standardized composition. FQs and SLIDs were frequently used. In the majority (six) of eight studies with available data bedaquiline was given for 6–6.5 months, however in two of eight studies the drug was also administered for longer time periods. Treatment outcomes were reported for 11 studies. Seven of those used WHO treatment outcomes, while three based the outcome assessment primarily on culture conversion.^40^ For nearly all studies with available outcome data the frequency of favourable outcomes was above 50% (10 of 11 studies), while almost half reported a frequency above 80% (5 of 11 studies). Detailed information on treatment regimens, bedaquiline exposure and outcomes can be found in Table 2.

**Table 2.** Treatment regimens, bedaquiline exposure and outcomes

Study	BDQ cohort	Regimen and drugs with high bactericidal activity	BDQ exposure	Outcomes^a^	Outcome definition and assessment
Conradie *et al*. 2020^57^	109	Standardized (BPaL) PTM (109/109), LZD (109/109)	6.5 months^b^	FV: 89.9% (98/109) UFV: 10.1% (11/109; 2 failures, 1 LTFU, 1 WOC, 7 deaths)	FV: Resolution of clinical disease, culture neg. and no UFV outcome at 6 months UFV: failure/relapse during follow-up (6 months after EOT)
Ghodousi *et al*. 2019^16c^	30	Individualized N/A	N/A	FV: 56.7% (17/30; 17 cured) UFV: 30.0% (9/30; 7 failures, 2 deaths) 4 N/A	WHO treatment outcomes^d^
Liu *et al*. 2021^58^	277	Individualized N/A	6 months	At 6 months: FV: 91.0% (252/277; 252 CC) UFV: 3.6% (10/277; 10 culture non-conversion) 15 N/A	CC: 2 consecutive neg. cultures during 6 month treatment or if only 1 neg. culture at 6 months together with clinical and radiological improvement
Nimmo *et al*. 2020^18^	92	N/A N/A	N/A	FV: 76.1% (70/92) UFV: 23.9% (22/92)	CC (after 6 months) WHO treatment outcomes^d^
Nimmo *et al*. 2020^47^	207	N/A N/A	N/A	N/A	N/A
Diacon *et al*. 2014^59^	79	Individualized FQ (66/66), SLID (64/66)^e^	6 months	At 26 months: FV: 58.0% (38/66; 38 cured) UFV: 42.0% (28/66; 5 failures, 15 WOC, 8 deaths)^e^	CC (after 6 months) WHO treatment outcomes^d^ (after 26 months)
Pym *et al*. 2016^19^	233	Individualized LVX (102/205), MXF (29/205), OFX (185/205), GAT (1/205), SLID (154/205)^e^	6 months	FV: 62.4% (128/205; 125 cured, 3 completed) UFV: 37.6% (77/205; 32 failures, 31 LTFU, 14 deaths)^e^	CC: 2 consecutive neg. cultures not followed by a pos. culture during the first 6 months, in case of death/discontinuation and without CC, classification as failure WHO treatment outcomes^d^ after 30 months
Guglielmetti *et al*. 2017^60^	45	Individualized MXF (24/45), LVX (8/45), AMK (32/45), CM (3/45), LZD (43/45), IPM/AMC (28/45), MEM/AMC (2/45)	6 months (12/45) or prolonged (33/45)	FV: 80.0% (36/45; 23 cured, 2 completed, 11 N/A) UFV: 20.0% (9/45; 1 failure, 5 LTFU, 3 deaths)	WHO treatment outcomes^d^
Guglielmetti *et al*. 2018^61^	10	Individualized MXF (5/10), CM (1/10), AMK (6/10), LZD (7/10), IPM/AMC (5/10), DLM (10/10)	Individualized (2–28 months)	FV: 90.0% (9/10; 9 cured) UFV: 10.0% (1/10; 1 LTFU)	WHO treatment outcomes^d^
Diacon *et al*. 2012^24^	23	Individualized KAN (23/23), rest N/A	6 months	At 6 months: FV: 81.0% (17/21^f^; 17 culture neg.) UFV: 19.0% (4/21^f^; 4 LTFU)	FV: culture neg. at time of assessment UFV: culture pos. at time of assessment or discontinuation; after 6 and 26 months
Kempker *et al*. 2020^62^	64	Individualized LVX/MXF (25/64), CM/KAN (43/64), LZD (50/64), IPM/AMC (9/64)	6 months	FV: 73.4% (47/64; 42 cured, 5 completed) UFV: 26.6% (17/64; 1 failure, 16 LTFU)	CC (after 2 and 6 months) WHO treatment outcomes^d^ (EOT); LTFU patients were reclassified as UFV in case of pos. culture after LTFU or as FV in case of initial CC and a subsequent post-treatment neg. culture
Mokrousov *et al*. 2021^42^	11	Individualized LVX (8/11), MXF (4/11), CM (3/11), AMK (4/11), LZD (3/11)	N/A	FV: 36.5% (4/11; 4 completed) UFV: 63.5% (7/11; 5 failures, 2 LTFU)	FV: completed UFV: LTFU, failure^g^
Nimmo *et al*. 2020^63^	147	N/A N/A	N/A	N/A	N/A

AMC, amoxicillin/clavulanic acid; AMK, amikacin; BDQ, bedaquiline; BPaL, bedaquiline, pretomanid and linezolid; CC, culture conversion; CM, capreomycin; DLM, delamanid; EOT, end of treatment; FV, favourable outcome; GAT, gatifloxacin; IPM, imipenem; KAN, kanamycin; LVX, levofloxacin; LTFU, loss to follow-up; LZD, linezolid; MXF, moxifloxacin; MEM, meropenem; N/A, not available; neg., negative; OFX, ofloxacin; pos.: positive; PTM, pretomanid; UFV, unfavourable outcome; WOC, withdrawal of consent.

a Primarily clinical outcomes of the WHO (if reported).

b Optional treatment extension by 13 weeks in case of culture positivity.

c Complemented with data from Tahseen *et al*. 2021.^21^

d Following the ‘Definitions and reporting framework for tuberculosis – 2013 revision’ of WHO.^40^

e Data for the modified intention-to-treat population.

f Only 21 patients were included in the efficacy analysis with assignment of treatment outcomes.

g The authors only reported the outcomes efficient treatment (treatment completed), treatment default and failure.

### Frequency of ABR

The median number of patients treated with bedaquiline was 79 per study (IQR 30–147). Baseline bedaquiline DST was done for the full patient cohort in 3 of 13 studies. For 6 of 13 studies it was not clearly specified whether all patients had bedaquiline DST results, and in 4 of 13 studies it was not performed for every participant. Only 7 of 13 studies specified the number of patients for whom sequential bedaquiline DST could be executed.

The median proportion of participants with baseline phenotypic bedaquiline resistance was 2.8% (IQR 1.9%–3.3%) and 4.2% of patients (IQR 1.6%–5.9%) already exhibited genotypic bedaquiline resistance before treatment start. The median frequency of phenotypic ABR amounted to 2.2% (IQR 1.1%–4.6%) and 4.4% (IQR 1.8%–5.8%) for genotypic ABR. Phenotypic and genotypic ABR combined with unfavourable treatment outcomes occurred in 1.1% (IQR 0.5%–3.9%) and 2.9% (IQR 1.0%–18.6%), respectively (Table 3).

**Table 3.** Bedaquiline DST and frequency of baseline and acquired bedaquiline resistance

Study	BDQ cohort	Patients with BDQ DST		Frequency of baseline BDQ resistance, % (*n*/*N*)		Frequency of ABR, % (*n*/*N*)		Frequency of ABR with adverse outcome^a^, % (*n*/*N*)	
		baseline^b^	sequential	MIC^c^	RAV^d^	MIC^e,g^	RAV^f,g^	MIC^e,g^	RAV^f,g^
Conradie *et al*. 2020^57^	109	57	N/A	2.8 (3/109)	N/A	0.9 (1/106)	0.9 (1/109)	0.9 (1/106)	0.9 (1/109)
Ghodousi *et al*. 2019^16h^	30	30	30	3.3 (1/30)	0.0 (0/30)	27.6 (8/29)	36.7 (11/30)	20.7 (6/29)	23.3 (7/30)
Liu *et al*. 2021^58^	277	**277**	94	2.2 (6/277)	1.1 (3/277)	3.0 (8/271)	1.8 (5/274)	1.1 (3/271)	1.1 (3/274)
Nimmo *et al*. 2020^18^	92	92	N/A	3.3 (3^i^/92)	5.4 (5/92)	6.7 (6^i^/89)	5.8 (5/87)	5.6 (5/89)	4.6 (4/87)
Nimmo *et al*. 2020^47j^	207	**207**	N/A	1.9 (4^i^/207)	2.9 (6/207)	3.9 (8^i^/203)	4.0 (8/201)	N/A	N/A
Diacon *et al*. 2014^59^	79	**79**	10	N/A	N/A	1.3 (1/79)	0.0 (0^k^/79)	N/A	0.0 (0^k^/79)
Pym *et al*. 2016^19^	233	**233**	24	N/A	N/A	5.2 (12/233)	5.2 (12/233)	N/A	N/A
Guglielmetti *et al*. 2017^60^	45	22	N/A	0.0 (0/45)	N/A	2.2 (1/45)	N/A	2.2 (1/45)	N/A
Guglielmetti *et al*. 2018^61^	10	10	10	40.0 (4/10)	N/A	0.0 (0/6)	N/A	0.0 (0/6)	N/A
Diacon *et al*. 2012^24^	23	20	N/A	8.7 (2/23)	N/A	0.0 (0/21)	N/A	0.0 (0/21)	N/A
Kempker *et al*. 2020^62^	64	62	N/A	0.0 (0/64)	N/A	1.6 (1/64)	N/A	N/A	N/A
Mokrousov *et al*. 2021^42^	11	**11**	11	N/A	9.1 (1^l^/11)	N/A	50.0 (5^l^/10)	N/A	50.0 (5^l^/10)
Nimmo *et al*. 2020^63^	147	**147**	83	N/A	6.1 (9/147)	N/A	4.4 (6/138)	N/A	N/A

BDQ, bedaquiline; N/A, not available.

a Adverse outcomes were treatment failure, death and loss to follow-up.

b If part of the protocol and not otherwise reported, performance assumed for all treated with bedaquiline (in bold).

c Phenotypic baseline bedaquiline resistance (MIC-based) was defined as when the initial MIC was above certain thresholds (7H9/7H11: 0.25 mg/L; MGIT: 1 mg/L).

d Genotypic baseline bedaquiline resistance (RAV-based) was defined as when the initial genotyping showed an *Rv0678*, *atpE* or *pepQ* mutation.

e In patients initially susceptible to bedaquiline, phenotypic ABR (MIC-based) was defined as when the follow-up MIC was either above defined thresholds (7H9/7H11: 0.25 mg/L; in MGIT: 1 mg/L) or was increased at least 4-fold but not lower than 0.12 mg/L on 7H9/7H11 or 0.5 mg/L on MGIT.

f The appearance of any new *Rv0678*, *atpE* or *pepQ* mutations in sequential isolates was considered as genotypic ABR (RAV-based).

g Denominator is patients treated with bedaquiline without baseline bedaquiline resistance, if reported.

h Complemented with data from Tahseen *et al*. 2021.^21^

i MIC testing was only performed for isolates with RAVs. MIC data were not available for one patient with bedaquiline RAV.

j Complemented with data from Nimmo *et al.* 2020.^63^

k Only tested for *atpE* mutations.

l One mutation appeared after 13 days, possibly pre-existed.

### Subgroup analysis: individual patient data

The availability of individual data of patients with ABR was limited (Tables S3 and S4). Only a few studies provided information on individual resistance patterns, treatment composition and outcomes of patients with phenotypic and/or genotypic ABR. The majority of patients with ABR developed an unfavourable outcome (65.2% and 69.2% of those with phenotypic and genotypic ABR, respectively).

Among the participants with phenotypic ABR the majority presented with an advanced resistance pattern at treatment start (pre-XDR 34.8%, XDR 47.8%) and the average number of likely effective drugs in the treatment regimens was 5 (IQR 3–5). All patients received linezolid. Only for two patients (14.3%) an FQ and for one patient (7.1%) an SLID were part of the regimen. Patients with unfavourable outcomes were more likely to have advanced resistance patterns such as pre-XDR or XDR (94.0% versus 62.5% of patients with a favourable outcome). The average number of likely effective drugs and bedaquiline protection were similar between participants with favourable and unfavourable outcomes.

A comparable trend could be noticed for the patients with genotypic ABR. Advanced drug resistance was predominant (pre-XDR 26.9%, XDR 57.7%) and the average number of likely effective drugs also amounted to 5 (IQR 3–5.5). The regimen of 16 individuals (76.2%) contained linezolid, whereas only 3 (14.3%) received an FQ and only 5 (23.8%) an SLID. Also, drug-resistance patterns, average number of likely effective drugs and bedaquiline protection showed a similar distribution between patients with favourable and unfavourable outcomes (Table 4).

**Table 4.** Summary of individual data of patients with phenotypic or genotypic ABR

Study/Outcome	Patients with ABR^a,b^	Initial resistance pattern^c^			Number of likely effective drugs, median (IQR)	Bedaquiline protection			
		MDR	pre-XDR	XDR		FQ	SLID	LZD	no
**Phenotypic ABR** ^a^									
Ghodousi *et al*. 2019^16d^									
FV	25.0 (2/8)	50.0 (1/2)	0.0 (0/2)	50.0 (1/2)	4 (—)	50.0 (1/2)	0.0 (0/2)	100.0 (2/2)	0.0 (0/2)
UFV	75.0 (6/8)	16.7 (1/6)	66.6 (4/6)	16.7 (1/6)	4 (3–5)	16.7 (1/6)	16.7 (1/6)	100.0 (6/6)	0.0 (0/6)
Nimmo *et al*. 2020^18e^									
FV	16.7 (1/6)	0.0 (0/1)	0.0 (0/1)	100.0 (1/1)	6 (—)	0.0 (0/1)	0.0 (0/1)	100.0 (1/1)	0.0 (0/1)
UFV	83.3 (5/6)	0.0 (0/5)	20.0 (1/5)	80.0 (4/5)	5 (4–6)	0.0 (0/5)	0.0 (0/5)	100.0 (5/5)	0.0 (0/5)
Guglielmetti *et al*. 2017^60^									
FV	0.0 (0/1)	—	—	—	—	—	—	—	—
UFV	100.0 (1/1)	0.0 (0/1)	0.0 (0/1)	100.0 (1/1)	N/A	N/A	N/A	N/A	N/A
Liu *et al*. 2021^58^									
FV	62.5 (5/8)	40.0 (2/5)	40.0 (2/5)	20.0 (1/5)	N/A	N/A	N/A	N/A	N/A
UFV	37.5 (3/8)	0.0 (0/3)	33.3 (1/3)	66.7 (2/3)	N/A	N/A	N/A	N/A	N/A
Total									
FV	34.8 (8/23)	37.5 (3/8)	25.0 (2/8)	37.5 (3/8)	5 (—)	33.3 (1/3)	0.0 (0/3)	100.0 (3/3)	0.0 (0/3)
UFV	65.2 (15/23)	6.7 (1/15)	40.0 (6/15)	53.3 (8/15)	5 (3–5)	9.1 (1/11)	9.1 (1/11)	100.0 (11/11)	0.0 (0/11)
All	100.0 (23/23)	17.4 (4/23)	34.8 (8/23)	47.8 (11/23)	5 (3–5)	14.3 (2/14)	7.1 (1/14)	100.0 (14/14)	0.0 (0/14)
**Genotypic ABR** ^b^									
Ghodousi *et al*. 2019^16d^									
FV	45.5 (5/11)	20.0 (1/5)	40.0 (2/5)	40.0 (2/5)	5 (3–5.5)	20.0 (1/5)	20.0 (1/5)	80.0 (4/5)	0.0 (0/5)
UFV	54.5 (6/11)	33.3 (2/6)	50.0 (3/6)	16.7 (1/6)	5 (3–5)	33.3 (2/6)	33.3 (2/6)	100.0 (6/6)	0.0 (0/6)
Nimmo *et al*. 2020^18e^									
FV	20.0 (1/5)	0.0 (0/1)	0.0 (0/1)	100.0 (1/1)	6 (—)	0.0 (0/1)	0.0 (0/1)	100.0 (1/1)	0.0 (0/1)
UFV	80.0 (4/5)	0.0 (0/4)	0.0 (0/4)	100.0 (4/4)	5.5 (4.5–6)	0.0 (0/4)	0.0 (0/4)	100.0 (4/4)	0.0 (0/4)
Mokrousov *et al*. 2021^42^									
FV	0.0 (0/5)	—	—	—	—	—	—	—	—
UFV	100.0 (5/5)	0.0 (0/5)	20.0 (1/5)	80.0 (4/5)	3 (3–5)	0.0 (0/5)	20.0 (1/5)	20.0 (1/5)	60.0 (3/5)
Liu *et al*. 2021^58^									
FV	40.0 (2/5)	50.0 (1/2)	0.0 (0/2)	50.0 (1/2)	N/A	N/A	N/A	N/A	N/A
UFV	60.0 (3/5)	0.0 (0/3)	33.3 (1/3)	66.7 (2/3)	N/A	N/A	N/A	N/A	N/Aq
Total									
FV	30.8 (8/26)	25.0 (2/8)	25.0 (2/8)	50.0 (4/8)	5 (3–6)	16.7 (1/6)	16.7 (1/6)	83.3 (5/6)	0.0 (0/6)
UFV	69.2 (18/26)	11.1 (2/18)	27.8 (5/18)	61.1 (11/18)	5 (3–5)	13.3 (2/15)	26.7 (4/15)	73.3 (11/15)	20.0 (3/15)
All	100.0 (26/26)	15.4 (4/26)	26.9 (7/26)	57.7 (15/26)	5 (3–5.5)	14.3 (3/21)	23.8 (5/21)	76.2 (16/21)	14.3 (3/21)

Values shown are % (*n*/*N*) unless stated otherwise.

FV favourable; LZD linezolid; UFV, unfavourable.

a In patients initially susceptible to bedaquiline, phenotypic ABR (MIC-based) was defined as when the follow-up MIC was either above certain thresholds (7H9/7H11: 0.25 mg/L; MGIT: 1 mg/L) or was increased at least 4-fold but not lower than 0.12 mg/L on 7H9/7H11 or 0.5 mg/L on MGIT.

b The appearance of any new *Rv0678*, *atpE* or *pepQ* mutations in sequential isolates was considered to be genotypic ABR (RAV-based).

c Following the WHO 2020 definitions of resistance patterns. Pre-XDR was defined as MDR-TB with additional drug resistance to any FQ or SLID.^7^

d Complemented with data from Tahseen *et al*. 2021.^21^

e Complemented with data from Nimmo *et al* 2020.^63^

### Quality assessment

The 11 cohort studies included in this analysis were given between 4 and 6 stars using the Newcastle-Ottawa Scale, which resulted in an overall assessment of a medium risk of bias (Figure S1). None of them had a control arm as part of the study design. The RoB 2 tool assessing the risk of bias in the two randomized controlled trials resulted in some concerns regarding study quality, due to the possibility of missing outcome data (Figure S2).

## Discussion

The median frequencies of phenotypic and genotypic ABR amounted to 2.2% (IQR 1.1%–4.6%) and 4.4% (IQR 1.8%–5.8%), respectively. This is coherent with the results of a model estimation by Kunkel *et al*.,^41^ who simulated a mean ABR frequency of 5.88% when using bedaquiline without tight restrictions for all patients with MDR-TB. However, for the latter study the method used for the determination of bedaquiline resistance was not specified. Our findings illustrate that genotypic methods do not show the same estimate as phenotypic approaches.

Nevertheless, the frequency of ABR calculated by our study might be biased in both directions. In the Pakistan cohort only patients with delayed culture conversion on bedaquiline treatment were included, while other cohorts included all those with an initially positive culture, also when culture converted early during bedaquiline treatment. This might be one of the reasons why the ABR frequencies with 27.6% (phenotypic) and 36.7% (genotypic) seem relatively high in comparison.^16^ Also, in the cohort of Mokrousov and colleagues^42^ a considerably elevated genotypic ABR frequency of 50% was detected. Even though not declared as such, a preselected set of patients as well as the low level of bedaquiline protection because of concurrent resistance to important drugs could explain this finding. On the other hand, there are reasons to believe that the degree of ABR might be higher in reality compared with our findings. Many of the studies did not specify the number of sequential isolates obtained. It might be possible that patients with a failure to produce an adequate follow-up sample might have harboured undetected bedaquiline resistance mutations. And, as there are estimations that acquired drug resistance accounts for about 38.7% of incident MDR-TB cases in previously treated patients, these individuals might be at higher risk of complex resistance patterns in case of relapse.^14^ It comes together with a commonly insufficient time interval for sequential bedaquiline DST, as only 4 of 13 studies clearly specified an appropriate follow-up time (until 6 months after the end of bedaquiline treatment) in regard to this aspect.

Participants who were lost to follow-up were at particular risk for ABR, due to the long termination half-life of bedaquiline. Therefore, actions to improve adherence are of utmost importance to prevent the acquisition of drug resistance.^43^ One approach to do so is a comprehensive strategy that includes interventions promoting the provision of enablers, incentives, education and holistic care.^44^ Particularly, alternative methods of directly observed therapy (DOT) like community-based DOT or new digital health solutions such as eDOT, eLearning and the usage of mobile communication could support individuals to successfully complete treatment.^45,46^

The difference in the frequency of phenotypic and genotypic ABR does not seem surprising. It is known that not all RAVs confer resistance.^47^ For example, *Rv0678* mutations in the transcriptional repressor MmpR can only confer bedaquiline resistance if the efflux pump is still functional.^31^ Therefore, MIC data might more accurately identify relevant ABR. Ghodousi *et al*.^16^ expressed that dynamic monitoring of MICs might be a better predictor of ABR than testing at a single critical concentration, as MIC rises can be manifold but still remain subthreshold. However, in case of heteroresistance or mixed infections increases of MIC levels might lag behind, whereas certain genotypic methods such as whole genome sequencing might offer decisive advantages as they have the capability to reveal ABR at earlier stages.^48^

When addressing the issue of ABR some other studies are worth mentioning that did not meet the eligibility criteria of this review. More specifically, the exact number of patients treated with bedaquiline in the parent cohorts was unknown, making it impossible to obtain a denominator for the calculation of the ABR frequency. Andres *et al*.^49^ examined samples from 124 MDR-TB patients treated with either bedaquiline or clofazimine processed by a German reference laboratory. They identified seven patients with elevated bedaquiline MICs meeting resistance criteria. For three of these individuals bedaquiline resistant isolates were already present in the first isolate, for two the bedaquiline MIC increases above the critical concentration occurred during bedaquiline treatment, for two during clofazimine treatment and for one during bedaquiline and clofazimine treatment. Five of these patients harboured *Rv0678* and one *atpE* mutations. These findings illustrate the importance of conducting baseline bedaquiline DST. The group around Zimenkov^50^ further investigated the isolates from 24 patients with an elevated MIC (7H11: ≥0.06 mg/L) from a bigger cohort treated with bedaquiline and linezolid. Among the 17 patients with available pretreatment isolates, 2 carried *Rv0678* mutations, 1 *atpE* mutations and 1 both at baseline. Three of those expressed MICs above the critical concentration. All 13 patients with available sequential isolates, excluding 3 participants already showing bedaquiline resistance before treatment start, developed some form of bedaquiline resistance during treatment (sole MIC increase in 2 cases, sole *Rv0678* mutation in 2 cases, *Rv0678* mutation and MIC increase in 8 cases, *atpE* mutation and MIC increase in 1 case). Thus demonstrating again the predominant role of *Rv0678* over other mutations in clinical practice.^50^ Peretokina *et al*.^48^ examined 345 isolates of 181 bedaquiline-naive individuals as well as bedaquiline-treated patients with unfavourable outcomes and a selected set with favourable outcomes. Among the 147 bedaquiline-naive isolates 6 (4.1%) displayed MICs above the critical concentration and 8 (5.4%) exhibited RAVs (*Rv0678* and *atpE*). Among the 58 isolates from patients treated with bedaquiline for ≤90 days 6 (10.4%) displayed MICs above the critical concentration and 5 (8.6%) exhibited RAVs and among the 130 isolates from patients treated with bedaquiline for >90 days (127 with an adverse outcome) 95 (73.0%) displayed MICs above the critical concentration and 99 (76.2%) exhibited RAVs. However, as the parent cohort treated with bedaquiline was not clearly defined and for many patients numerous isolates were taken, it was not possible to calculate a frequency of ABR.^48^ Nonetheless, these findings demonstrate that ABR is more likely to become apparent after the first 3 months of treatment and is mostly associated with unfavourable treatment outcomes.

Only a subset of the included studies in this review reported individual data of patients who acquired bedaquiline resistance. The median number of likely effective drugs was five, which is more than the minimum of four likely effective drugs recommended by WHO.^7^ Hence, regimen composition including synergistic drug mechanisms might be more important than just adding a certain number of drugs. Van Deun *et al*.^23^ propose a core drug as the central part of a solid regimen, characterized by moderate to high bactericidal and sterilizing activity and without evidence for (cross-)resistance to core drugs used in previous regimens. Additionally, two drugs with (high) bactericidal activity and two with sterilizing activity should be used as so-called companion drugs.^23^

As explained earlier, patients with undetected FQ resistance are particularly at risk of developing ABR. According to Chiang *et al*.^51^ the WHO recommendation to perform pretreatment FQ DST is not feasible in the majority of settings, as it is either not available for many patients or results only arrive after ABR might have already occurred. However, in case of heteroresistance with mutants present in less than 1% of the mycobacterial population, phenotypic DST and even newer genotypic DST methods available in high-resource settings (e.g. targeted next-generation sequencing) will not be able to detect FQ resistance before treatment initiation.^52^ And, it seems likely that in populations with a considerable level of FQ resistance, in some of those patients with susceptibility at baseline, FQ resistant mutants would multiply before the onset of bedaquiline’s bactericidal activity and emerge above the 1% threshold.^53^ This shows that pretreatment testing might not be sufficient to avoid the emergence of ABR.

Chiang *et al*.^51^ proposed that the addition of linezolid to short treatment regimens may be a temporary option before obtaining FQ DST results. However, in our subgroup analysis regimens of patients with ABR predominantly included linezolid, which supports scepticism that its resistance protecting activity might be limited.^18,21^ Recently published data from Bangladesh support the assumption that its early killing effect is too little to sufficiently protect the regimen’s core drug against the development of resistant mutants.^54^ Furthermore, Chiang *et al*.^51^ propose to consider the utilization of injectable-containing short MDR-TB regimens again. Adapted administration intervals and strict audiometric monitoring could reduce the occurrence of adverse effects remarkably. Backed by data from DR-TB patients in Pakistan, this view is shared by Tahseen *et al*.,^21^ who have expressed concerns about the rationale of the WHO for phasing out the injectable drugs. Additionally, it could be shown that the replacement of the FQ by bedaquiline using the Bangladesh regimen (including the injectable kanamycin) in case of high-level FQ resistance results in higher rates of culture conversion and relapse-free cure, thus demonstrating that bedaquiline but not linezolid can act as core drug for the treatment of patients with FQ-resistant RR-TB.^54^

Past clofazimine exposure can result in the emergence of some *Rv0678* mutations with cross-resistance to bedaquiline, about a third of clofazimine resistant isolates are also bedaquiline-resistant.^10,55,56^ As a WHO classified group B drug, clofazimine is essential for the treatment of RR-TB and widely used. Various studies have demonstrated its positive effect on treatment success, time to culture conversion and cavity closure rate.^55^ To not undermine the effective use of bedaquiline in the future, clinicians should assess patients with a history of clofazimine treatment in the past, especially after treatment failure, and prioritize those individuals for bedaquiline DST before treatment initiation in case of limited resources.

Yet, should we be more prudent with the indication of bedaquiline, maybe restricting its use to patients with more advanced resistance patterns? Based on a meta-analysis of the effect of drugs (but not of regimens) Kunkel and colleagues^41^ developed a mathematical decision model to estimate the impact of providing bedaquiline to different subcategories of DR-TB patients. They found that by limiting bedaquiline access solely to patients with more advanced resistance patterns, the risk of bedaquiline resistance might be reduced, but thus the risk of resistance to other drugs (e.g. FQs) would be maximized. Besides, they stated that a more liberal use of bedaquiline could lower overall transmission of DR-TB and improve the number and outcomes of secondary cases. As a consequence, they concluded that bedaquiline should be available for all patients with MDR-TB.^41^ However, the model was based on the former definitions of pre-XDR and XDR-TB and whether their findings on the protective effect of bedaquiline on overall resistance acquisition are reproducible in the real world would need to be confirmed. Other authors argue that safeguarding bedaquiline for third-line TB treatment secures effective treatment options for the bulk of patients, including those with failure or relapse after a first MDR/RR-TB treatment regimen.^23^

Lastly, Tahseen *et al*.^21^ gave us an important reminder. They illustrated that acquired rifampicin resistance was detected initially in just about 0.1% of patients. However, merely one decade later the prevalence of primary rifampicin resistance rose to 2%–15% in some settings. Our assessed occurrence of ABR was manifold higher, urging public health professionals and clinicians for caution in using this novel treatment agent.

### Limitations

Our findings should be regarded in light of certain limitations. The analysis of general characteristics revealed a large variety of study populations. As treatment regimens were mostly individualized, it was not possible to assess ABR by regimen. Retrieved study data were partly incomplete and the method of assessment often not standardized. Therefore, we refrained from performing a meta-analysis. The determined quality of the included studies was mediocre, raising some concerns about a possible risk of bias in and between studies. Moreover, only the initial DST results and treatment regimens were considered, disregarding that DR-TB management is a dynamic process. Also, studies solely reporting on bedaquiline resistance were not part of this review, even though they might add valuable information in regard to characteristics of patients with ABR. We did not summarize data on non-adherence, a potential cause of resistance amplification, as individual studies did either not report data on it or used different indicators of adherence.

### Conclusions

Our findings demonstrate the relevance of ABR during bedaquiline-containing treatment of patients with DR-TB. Regimens should be constructed considering the activity of the used antimicrobial agents with a focus on the protection of bedaquiline, not merely relying on the number of administered drugs.^23^ Maximal efforts must be made by stakeholders to ensure the availability of phenotypic and genotypic DST methods for FQs, thus enabling programmes to provide an effective, less toxic regimen with higher probability of favourable outcomes. Bedaquiline should only be used as part of a solid treatment regimen, which yet has to be developed considering the apparent inadequacy of the currently recommended priority drugs, besides hard to exclude FQ resistance. Adapted treatment compositions should be examined with the aim of protecting bedaquiline, particularly in the first week before the full development of its bactericidal activity with FQ-resistant mutants poorly covered, not shying away from nowadays less used medications such as SLIDs. Surveillance of drug resistance is crucial to assess the incidence and prevalence of bedaquiline resistance to guide its use, especially in locations where DST capacity is limited. The availability of reliable and rapid bedaquiline DST needs to be extended, also in order to identify and protect patients with baseline bedaquiline resistance from adverse treatment outcomes. Standardized ABR definitions are essential and a comprehensive register of bedaquiline RAVs is necessary to group and correlate them according to their level of conferred phenotypic resistance. Studies including bedaquiline should follow predefined protocols for reporting and address the frequency of bedaquiline resistance in a standardized manner.

## Supplementary Material

dlac029_Supplementary_Data

## Supplementary data

Tables S1 to S4 and Figures S1 and S2 are available as Supplementary data at *JAC-AMR* Online.
