# Supplementary material for: Acquired bedaquiline resistance during the treatment of drug-resistant tuberculosis: a systematic review
Source: JAC Antimicrob Resist. 2022 Mar 29;4(2):dlac029. doi: 10.1093/jacamr/dlac029 (PMC8963286; doi:10.1093/jacamr/dlac029)
Supplement: dlac029_Supplementary_Data [file dlac029_supplementary_data.docx]

# Supplementary data

**Table S1. Search strategy and study selection.**

| **1. Research question: PICO approach** | |
| --- | --- |
| **Person (P)** | People with drug-resistant tuberculosis |
| **Intervention (I)** | Treatment with bedaquiline-containing regimens |
| **Comparator (C)** | - |
| **Outcome (O)** | Frequency of acquired bedaquiline resistance |
| **2. Objectives** | |
| **Primary** | Estimation of the frequency of acquired bedaquiline resistance |
| **Secondary** | Assessment of trial characteristics, treatment regimens and treatment outcomes  Analysis of patient features with acquired bedaquiline resistance (e.g. resistance profile, treatment regimen and duration) |
| **3. Search terms** | |
| **CENTRAL** | ‘bedaquiline AND resistan*’ (full text) |
| **PubMed** | ‘bedaquiline resistan*’ (full text, automated term mapping) |
| **Embase** | ‘”bedaquiline NEAR/10 resistan*”’ (full text) |
| **4. Study selection** | |
| **Inclusion**  **criteria** | English language  Only human participants  Only original studies  *Mycobacterium tuberculosis*  Bedaquiline-containing regimens  Sequential bedaquiline drug-susceptibility testing  Not solely reporting on bedaquiline resistance |
| **Exclusion criteria** | Article not available in English  Animal studies  No original studies (e.g. reviews)  No *Mycobacterium tuberculosis* (e.g. non-tuberculous mycobacteria)  No bedaquiline-containing regimens  No clear denominator for number of patients treated with a bedaquiline-containing regimen  No sequential bedaquiline drug-susceptibility testing  Average treatment duration < 6 months  Solely reporting on bedaquiline resistance (e.g. case reports) |

**Table S2. Calculation of the frequency of acquired bedaquiline resistance.**

| **Measure** | **Numerator** | **Denominator** |
| --- | --- | --- |
| **Phenotypic ABR** | Number of patients with  **MIC‑based^a^ ABR** | Number of patients **treated with BDQ** and  **without baseline MIC-based^b^ BDQ resistance** |
| **Genotypic ABR** | Number of patients with  **RAV‑based^c^ ABR** | Number of patients **treated with BDQ** and  **without baseline RAV‑based^d^ BDQ resistance** |
| **Phenotypic ABR with adverse outcome^e^** | Number of patients with  **MIC‑based^a^ ABR** and **adverse outcome^e^** | Number of patients **treated with BDQ** and  **without baseline MIC‑based^b^ BDQ resistance** |
| **Genotypic ABR with adverse outcome^e^** | Number of patients with  **RAV‑based^c^ ABR** and **adverse outcome^e^** | Number of patients **treated with BDQ** and  **without baseline RAV‑based^d^ BDQ resistance** |

7H9/7H11: Middlebrook 7H9 broth or 7H11 agar base, ABR: Acquired bedaquiline resistance, BDQ: Bedaquiline, MGIT: Mycobacteria growth indicator tube, RAV: Resistance-associated variant

^a^ In patients initially susceptible to BDQ, phenotypic ABR (MIC-based) was defined when the follow-up MIC was either above defined thresholds (7H9/7H11: 0,25 mg/L; in MGIT: 1 mg/L) or was increased at least 4-fold but not lower than 0.12 mg/L on 7H9/7H11 or 0.5 mg/L on MGIT

^b^ Phenotypic baseline BDQ resistance (MIC-based) was defined when the initial MIC was above certain thresholds (7H9/7H11: 0.25 mg/L; MGIT: 1 mg/L)

^c^ The appearance of any new *Rv0678*, *atpE* or *pepQ* mutations in sequential isolates was considered as genotypic ABR (RAV-based)

^d^ Genotypic baseline BDQ resistance (RAV-based) was defined when the initial genotyping showed a *Rv0678*, *atpE* or *pepQ* mutation

^e^ Adverse outcomes were treatment failure, death and loss to follow-up

| Study | Patients with ABR | | Mutations (type, number and percentage) | | | | Median number of acquired mutations per patient (range) | | | | Median month when ABR was detected (IQR)^3^ | Median change in MIC (IQR) | Median MIC when ABR was detected in mg/L (IQR) | Outcomes of patients with ABR | |
| --- | --- | --- | --- | --- | --- | --- | --- | --- | --- | --- | --- | --- | --- | --- | --- |
|  | **MIC^1^** | **RAV^2^** | **Rv0678** | **atpE** | **pepQ** | **Rv1979c** | **Rv0678** | **atpE** | **pepQ** | **Rv1979c** |  |  |  | **MIC^1^** | **RAV^2^** |
| Conradie et al. 2020 ^36^ | 1 | 1 | 1/1  (100.0 %) | N/A | N/A | N/A | 1  (1) | N/A | N/A | N/A | N/A | MGIT: 8x  (-) | MGIT: 4  (-) | 1 FL | 1 FL |
| Ghodousi et al. 2019^a^ ^14^ | 8 | 11 | 8/15  (53.3 %) | 0/15  (0.0 %) | 6/15  (40.0 %) | 1/15  (6.7 %) | 1  (1-2) | 0  (0) | 1  (1-2) | 1  (0) | 4  (2.5-5.5) | 7H11: 4x (1.75-8x) | 7H11: 0.185 (0.06-0.3125)^b^ | 2 CU, 5 FL,  1 D | 5 CU, 6 FL |
| Liu et al. 2020 ^37^ | 8 | 5 | 5/5  (100.0 %) | 0/5  (0 %) | 0/5  (0.0 %) | 0/5  (0 %) | 1  (1) | 0  (0) | 0  (0) | 0  (0) | N/A | 7H9: 6x  (4-8x) | 7H11 : 0.25 (0.12-0.25) | 5 CC,  3 CNC | 2 CC, 3 CNC |
| Nimmo et al. 2020a^c^ ^16^ | 6 | 5 | 6/12  (50.0 %) | 0/12  (0.0 %) | 0/12  (0.0 %) | 6/12  (50.0 %) | 1  (1-9) | 0  (0) | 0  (0) | 1  (1-2) | 4  (2.5-5.5) | 7H11: 12x  (8-32x) | 7H11: 0.5  (0.3125-0.875) | 1 SU, 1 FL,  4 D | 1 SU, 1 FL, 3 D |
| Nimmo et al. 2020b ^38^ | 9 | 8 | 9/17  (52.9 %) | 0/17  (0.0 %) | 0/17  (0 %) | 8/17  (47.1 %) | 1  (1-9) | 0  (0) | 0  (0) | 1  (1-2) | 4  (2-6) | 7H11: 8x  (8-20x)^d^ | 7H11: 0.5  (0.25-0.625)^d^ | 1 SU, 1 FL,  4 D, 2 N/A | 1 SU, 1 FL, 3 D, 3 N/A |
| Diacon et al. 2014 ^39^ | 1 | 0 | N/A | 0/1  (0.0 %) | N/A | N/A | N/A | N/A | N/A | N/A | N/A | 7H11: 4x  (-) | N/A | N/A | N/A |
| Pym et al. 2016 ^17^ | 12 | 12 | 12/12  (100.0 %) | 0/12  (0.0 %) | N/A | N/A | N/A | N/A | N/A | N/A | N/A | N/A | N/A | 5 SU, 3 FL,  4 N/A | 5 SU, 3 FL,  4 N/A |
| Guglielmetti et al. 2017 ^40^ | 1 | N/A | N/A | N/A | N/A | N/A | N/A | N/A | N/A | N/A | 14  (-) | N/A | N/A | 1 FL | N/A |
| Guglielmetti et al. 2018 ^41^ | 0 | N/A | N/A | N/A | N/A | N/A | N/A | N/A | N/A | N/A | N/A | N/A | N/A | 0 | N/A |
| Diacon et al. 2012 ^22^ | 0 | N/A | N/A | N/A | N/A | N/A | N/A | N/A | N/A | N/A | N/A | N/A | N/A | 0 | N/A |
| Kempker et al. 2020 ^42^ | 1 | N/A | N/A | N/A | N/A | N/A | N/A | N/A | N/A | N/A | N/A | 7H9: 8x  (-) | 7H9: 0.03  (-) | N/A | N/A |
| Mokrousov et al. 2020 ^43^ | N/A | 5 | 4/5  (80.0 %) | 1/5  (20.0 %) | 0/5  (0.0 %) | 0/5  (0.0 %) | 1.5  (1-4) | 1  (1) | 0  (0) | 0  (0) | N/A | N/A | N/A | N/A | 4 FL,  1 LTFU |
| Nimmo et al. 2020c ^44^ | N/A | 6 | 6/6  (100.0 %) | 0/6  (0.0 %) | 0/6  (0.0 %) | N/A | 1.5  (1-8) | 0  (0) | 0  (0) | N/A | 5  (2-6) | N/A | N/A | N/A | N/A |

**Table S3. Summary of characteristics of individual patients.**

7H9/7H11: Middlebrook 7H9 broth or 7H11 agar base, ABR: Acquired bedaquiline resistance, CC: Culture converted, CNC: Culture non-conversion, CU: Cured, D: Death, FL: Failure, LTFU: Loss to follow-up, MGIT: Mycobacteria growth indicator tube, N/A: Not available, RAV: Resistance-associated variant, SU: Successful

^1^ In patients initially susceptible to BDQ, phenotypic ABR (MIC-based) was defined when the follow-up MIC was either above certain thresholds (7H9/7H11: 0.25 mg/L; MGIT: 1 mg/L) or was increased at least 4-fold but not lower than 0.12 mg/L on 7H9/7H11 or 0.5 mg/L on MGIT

^2^ The appearance of any new *Rv0678*, *atpE* or *pepQ* mutations in sequential isolates was considered as genotypic ABR (RAV-based)

^3^ Primarily based on MIC evolution. If no MIC data was available, based on the appearance of *Rv0678*, *atpE* or *pepQ* mutations

^a^ Complemented with data from Tahseen et al. 2020 ^19^

^b^ Not available for 2 patients

^c^ Complemented with data from Nimmo et al. 2020b ^38^

^d^ MIC data was not available for one patient with BDQ RAV

**Table S4. Individual treatment regimens, DST results and treatment outcomes in patients with acquired BDQ resistance (if reported).**

| **Study** | **Pat.** | **TBH** | **BDQ-containing regimen^1^** | **Initial resistance^2^** | **DST profile** | **Number of LEDs^3^** | **BDQ Protection^4^** | **BDQ mutation and month** | | **Change in MIC in mg/L^5^** | **OC** |
| --- | --- | --- | --- | --- | --- | --- | --- | --- | --- | --- | --- |
| **Ghodousi et al. 2019^a^ ^14^** | 1 | N/A | **BDQ, LZD, ETO, CFZ, Z**, MFX, AM, E | MFX, AM, E | pre-XDR | 5 | LZD | Rv0678: | M4 | 7H11:  0.12 → 0.5 | FL |
|  | 2 | N/A | **BDQ, LFX, LZD, ETO, CFZ**, CS, *AM, PAS*, Z | Z | MDR | 5 | FQ, LZD | Rv0678:  Rv1979c: | M5  M0 | 7H11:  0.03 → 0.5 | CU |
|  | 3 | N/A | **BDQ, MFX, CM, LZD, CFZ**, *CS, PAS*, ETO, E, Z | ETO, E, Z | MDR | 5 | FQ, SLID, LZD | Rv0678:  pepQ: | M6  N/A | 7H11:  0.06 → 0.25 | FL |
|  | 4 | N/A | **BDQ, LZD, CFZ,** *CS, PAS*, MFX, CM, ETO, Z | MFX, CM, ETO, Z | XDR | 3 | LZD | Rv0678: | M7 | 7H11:  0.03 → 0.25 | CU |
|  | 5 | N/A | **BDQ, LZD, CFZ**, *CS*, LFX, ETO, E, H, Z | LFX, ETO, E, H, Z | pre-XDR | 3 | LZD | Rv0678: | M3 | 7H11:  0.03 → 0.12 | FL |
|  | 6 | N/A | **BDQ, LZD, DLM, ETO, CFZ**, LFX, H, Z | LFX, H, Z | pre-XDR | 5 | LZD | Rv0678: | M1 | 7H11:  0.03 → 0.5 | FL |
|  | 7 | N/A | **BDQ, LZD, CFZ**, *CS, AM, PAS* | MFX, CM, ETO, Z | XDR | 3 | LZD | Rv0678:  pepQ: | M5  N/A | 7H11:  0.03 → 0.12 | FL |
|  | 8 | N/A | **BDQ, LZD, CFZ**, *CS*, LFX, ETO, H, Z | LFX, ETO, H, Z | pre-XDR | 3 | LZD | WT |  | 7H11:  0.03 → 0.25 | D |
|  | 9 | N/A | **BDQ, LZD, CFZ**, *CS, PAS*, LFX, ETO, E, Z | LFX, ETO, E, Z | pre-XDR | 3 | LZD | pepQ: | M9 | 7H11:  0.12 → 0.06 | CU |
|  | 10 | N/A | **BDQ, CM, ETO, CFZ, Z**, MFX, E | MFX, E | pre-XDR | 5 | SLID | pepQ: | M1 | 7H11:  0.06 → 0.06 | CU |
|  | 11 | N/A | **BDQ, LZD, ETO, CFZ, PAS, Z**, *CS*, LFX, CM | LFX, CM | XDR | 6 | LZD | Rv0678:  pepQ: | M2 N/A | 7H11:  0.008 → 0.016 | CU |
|  | 12 | N/A | **BDQ, MFX, CM, LZD, CFZ, DLM, ETO, CS, PAS**, *AM, CLR* | E | MDR | 9 | FQ, SLID, LZD | pepQ: | M4 | 7H11:  0.25 → 0.03 | FL |
| **Nimmo et al. 2020a^b^ ^16^** | 1 | No | **BDQ, LZD, CFZ, TZ, PAS, Z,** FQ | FQ, SLID, H, R | XDR | 6 | LZD | Rv0678: Rv1979c: | M4  M0 | 7H11:  0.06 → 1.0 | D |
|  | 2 | Yes | **BDQ, ETO, LZD, CFZ, TZ, PAS Z, E,** FQ | FQ, SLID, H, R | XDR | 6 | LZD | Rv0678:  Rv1979c: | M4  M0 | 7H11:  0.03 → 1.0 | SU |
|  | 3 | Yes | **BDQ, LZD, TZ, PAS, Z,** FQ, CFZ, ETO, E | FQ, SLID, CFZ, ETO, H, R, E | XDR | 4 | LZD | Rv0678:  Rv1979c: | M6  M0 | 7H11:  0.06 → 0.5 | FL |
|  | 4 | Yes | **BDQ, LZD, CFZ, TZ, PAS, DLM** | FQ, SLID, H, R | XDR | 6 | LZD | Rv0678: | M6 | 7H11:  0.03 → 0.25 | D |
|  | 5 | No | **BDQ, CFZ, LZD, TZ, PAS, Z,** FQ, ETO | FQ, SLID, ETO, H, R | XDR | 5 | LZD | Rv0678: | M2 | 7H11:  0.03 → 1.0 | D |
|  | 6 | Yes | **LZD, TZ, PAS, IMP/A-C,** BDQ, FQ, CFZ, Z | BDQ, FQ, CFZ, H, R, Z | pre-XDR | 4 | LZD | Rv0678: | M0 | 7H11:  0.03 → 0.25 | D |
| **Gugliel-metti et al. 2017 ^40^** | 1 | N/A | **BDQ, LZD, CFZ, AM, ETO, PAS** | N/A | XDR | N/A | No | N/A |  | N/A | FL |
| **Mokrou-sov et al. 2020 ^43^** | 1 | Yes | **BDQ, CM, TZ, PCZ, Z,** MFX | OFX, ETO, PAS, H, R, S, E | pre-XDR | 5 | SLID | Rv0678: | N/A | N/A | FL |
|  | 2 | Yes | **BDQ, PAS, PCZ,** LFX, AM, Z | OFX, KM, CM, CS, PTO, H, R, S, Z, E | XDR | 3 | No | atpE: | N/A | N/A | LTFU |
|  | 3 | N/A | **BDQ, LZD, CS, E, PCZ,** LFX | OFX, KM, CM, AM, PTO, H, R, S, Z | XDR | 5 | LZD | Rv0678: | N/A | N/A | FL |
|  | 4 | Yes | **BDQ, CFZ, PCZ,** LFX, Z | OFX, KM, AM, PAS, CS, ETO, H, R, S, E, Z | XDR | 3 | No | Rv0678: | N/A | N/A | FL |
|  | 5 | Yes | **BDQ, PAS, Z,** LFX, AM | OFX, KM, ETO, CS, H, R, S, E | XDR | 3 | No | Rv0678: | N/A | N/A | FL |
| **Nimmo et al. 2020c ^44^** | 1 | N/A | **BDQ, LFX, CFZ, ETO, E, Z, Hh** | R | RR | 7 | FQ | Rv0678: | M0 | N/A | N/A |
|  | 2 | N/A | **BDQ, TZ, PAS,** LFX, CFZ, Z | FQ, SLID, R, H, ETO, Z, CFZ, S | XDR | 3 | No | Rv0678: | M4 | N/A | N/A |
|  | 3 | N/A | **BDQ, TZ, PAS,** LFX, CFZ, ETO, E, Z | FQ, SLID, H, R, Z, E, CFZ, ETO, S | XDR | 3 | No | Rv0678: | M6 | N/A | N/A |
|  | 4 | N/A | **BDQ, DLM, LZD, CFZ, TZ, PAS** | FQ, SLID, R, H, Z, E | XDR | 6 | LZD | Rv0678: | M6 | N/A | N/A |
|  | 5 | N/A | **BDQ, LZD, TZ, PAS,** LFX, CFZ, ETO, Z | FQ, SLID, R, H, Z, E, CFZ, ETO | XDR | 4 | LZD | Rv0678: | M2 | N/A | N/A |
|  | 6 | N/A | **BDQ, LFX, CFZ, E, Z, Hh** | R | RR | 6 | FQ | Rv0678: | M6 | N/A | N/A |
| **Liu et al. 2020** ^37^ | 1 | Yes | N/A | N/A | MDR | N/A | N/A | WT | N/A | 7H9:  0.03 → 0.5 | CC |
|  | 2 | Yes | N/A | N/A | XDR | N/A | N/A | Rv0678 | N/A | 7H9:  0.03 → 0.25 | CNC |
|  | 3 | Yes | N/A | N/A | XDR | N/A | N/A | Rv0678 | N/A | 7H9:  0.06 → 0.25 | CNC |
|  | 4 | Yes | N/A | N/A | Pre-XDR | N/A | N/A | Rv0678 | N/A | 7H9:  0.03 → 0.25 | CNC |
|  | 5 | No | N/A | N/A | Pre-XDR | N/A | N/A | WT | N/A | 7H9:  0.03 → 0.25 | CC |
|  | 6 | Yes | N/A | N/A | XDR | N/A | N/A | Rv0678 | N/A | 7H9:  0.03 → 0.12 | CC |
|  | 7 | Yes | N/A | N/A | MDR | N/A | N/A | Rv0678 | N/A | 7H9:  0.03 → 0.12 | CC |
|  | 8 | Yes | N/A | N/A | Pre-XDR | N/A | N/A | WT | N/A | 7H9:  0.03 → 0.12 | CC |
|  | 1 | Yes | N/A | N/A | MDR | N/A | N/A | WT | N/A | 7H9:  0.03 → 0.5 | CC |

7H11: Middlebrook 7H11 agar base, AM: Amikacin, BDQ: Bedaquiline, CM: Capreomycin, CFZ; Clofazimine, CLR: Clarithromycin, CS: Cycloserine, CU: Cured, D: Death, DLM: Delamanid, DST: Drug-susceptibility testing, E: Ethambutol, ETO: Ethionamide, FL: Failure, FQ: Fluoroquinolone, Hh: Isoniazid high dose, IMP/A‑C: Imipenem(Amoxicillin-Clavulanic acid), KM: Kanamycin, LEDs: Likely effective drugs, LFX: Levofloxacin, LTFU: Loss to follow-up, LZD: Linezolid, MFX: Moxifloxacin, OC: Outcome, OFX: Ofloxacin, PAS: Para-aminosalicylic acid, PTM: Pretomanid, pre‑XDR: pre-extensively drug-resistance, Pat.: Patient, PCZ: Perchlozone, PTO: Prothionamide, RR: Rifampicin-resistant, SLID: Second-line injectable drug, SU: Successful, TBH: TB History, TZ: Terizidone, WT: Wild type, Z: Pyrazinamide

^1^ When starting BDQ; drugs previously used in *italic*, drugs with known initial resistance are underscored

^2^ Following the WHO 2020 definitions of resistance patterns. Pre-XDR was defined as MDR-TB with additional drug resistance to any FQ or SLID ^6^

^3^ Includes those drugs with data on initial susceptibility; if no DST data were reported for a specific drug, drugs were considered to be likely effective

^4^ By a FQ, SLID or LZD; if the drug was included in the regimen when BDQ was started and also likely effective

^5^ Primarily based on MIC evolution. If no MIC data was available, based on the appearance of any new *Rv0678*, *atpE* or *pepQ* mutations

^a^ Complemented with data from Tahseen et al. 2020 ^19^

^b^ Complemented with data from Nimmo et al. 2020b ^38^

Inspired by Tahseen et al. 2020 ^19^


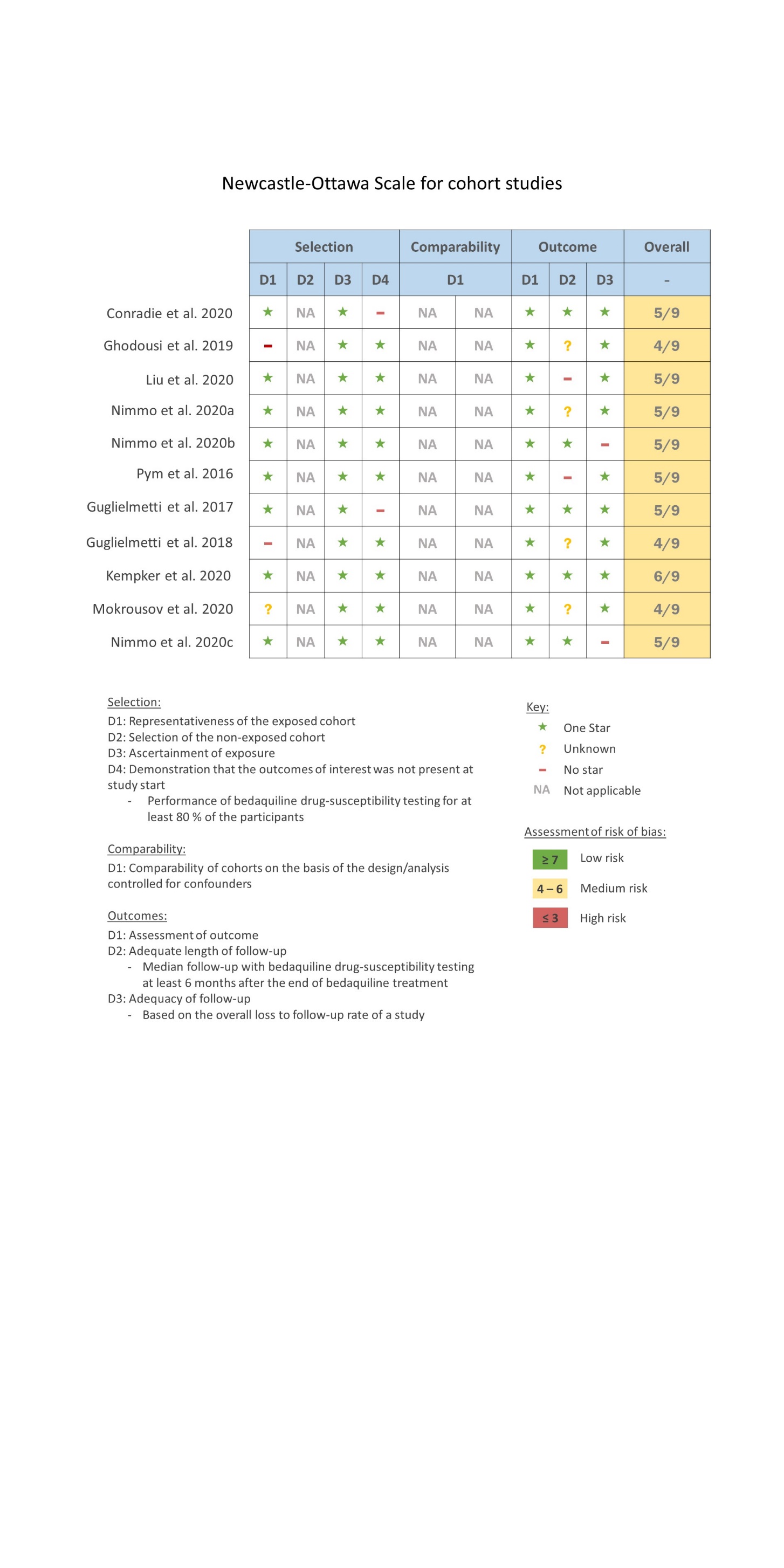


**Figure S1.** **Qualitative assessment with the Newcastle-Ottawa Scale for cohort studies.**


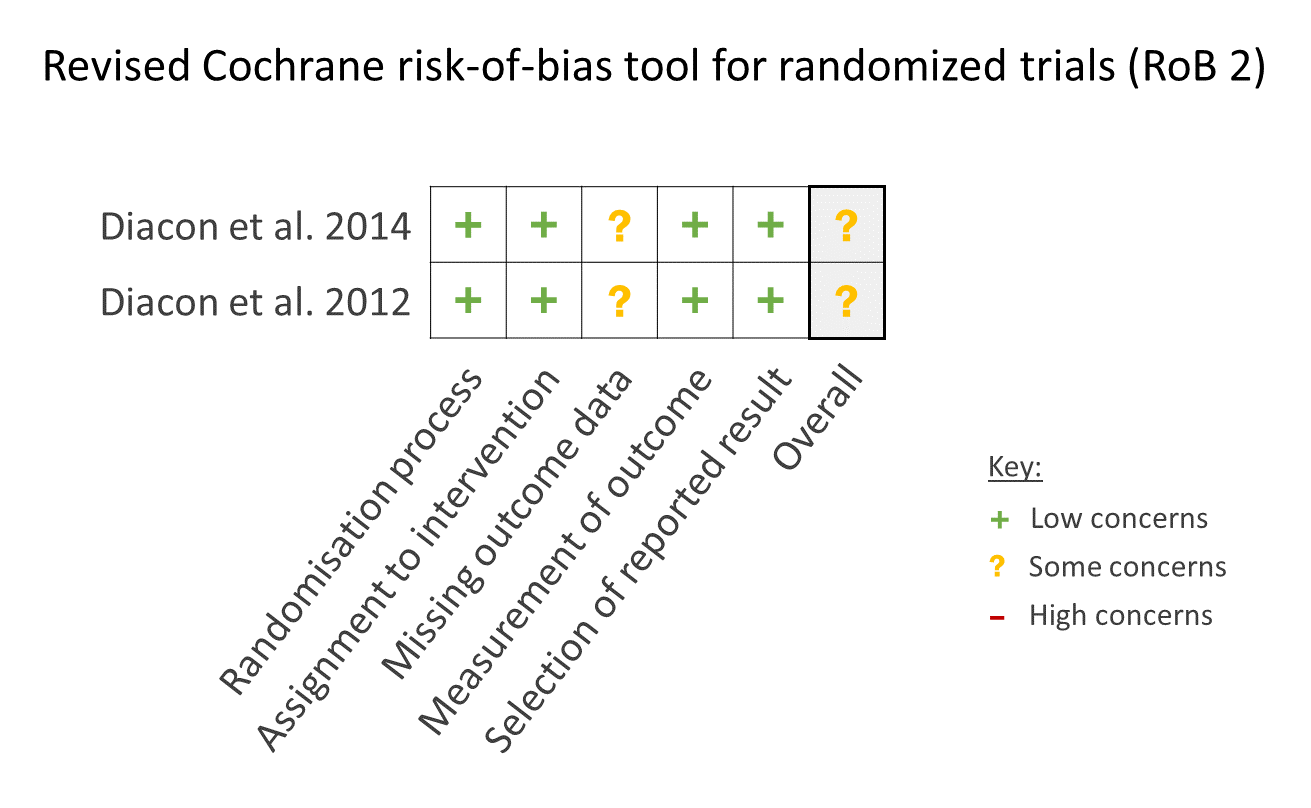


**Figure S2.** **Qualitative assessment with the Revised Cochrane risk-of-bias tool for randomized trials (RoB 2).**
